# Diagnostic Value of the Combined Detection of Microbiota, Multiple Inflammation-Related Indicators, Serum Lipid Indices, and Tumour Markers in Colorectal Polyps: A Case-Control Study

**DOI:** 10.7150/ijms.108819

**Published:** 2025-05-30

**Authors:** Limin Zhang, Guoxiu Xiao, Duyao Su, Xun Wang, Cuiting Lv, Chunchun Li, Mingsheng Fu, Liang Song

**Affiliations:** 1 Department of Stomatology, Shanghai Fifth People's Hospital, Fudan University, Shanghai 200240, China.; 2 Center of Community-based Health Research, Fudan University, Shanghai 200240, China.; 3 Central Laboratory, Shanghai Fifth People's Hospital, Fudan University, Shanghai 200240, China.; 4 Department of gastroenterology, Shanghai Fifth People's Hospital, Fudan University, Shanghai 200240, China.

**Keywords:** Colorectal polyp, Microorganism, Neutrophil-to-lymphocyte-count ratio, Triglyceride, Low-Density Lipoprotein Cholesterol, Carcinoembryonic Antigen

## Abstract

**Purpose:** Colorectal polyps have few clinical symptoms, and related tumor markers are unclear; therefore, developing a simple and economical tumor detection index for auxiliary diagnosis is necessary. We aimed to investigate differences in salivary and fecal microbiota, inflammation-related indicators, serum lipid indices, and tumor markers between patients with colorectal polyps and healthy controls, to identify novel non-invasive biomarkers for colorectal polyps.

**Patients and methods:** This case-control study enrolled 47 patients with colorectal polyps and 59 age- and sex-matched healthy controls between 13 May 2022 and 20 November 2023. From each participant, we collected salivary and fecal samples, fasting venous blood samples, polyp tissues, and normal intestinal tissues. We then evaluated the diagnostic performance of multiple markers, including salivary and fecal microbiota, routine blood tests, blood lipids, serum tumor markers, and the NOD-like receptor protein 3 (NLRP3) inflammasome, both individually and in combination. The assessment was based on metrics such as the Youden index, sensitivity, and specificity.

**Results:** There were statistically significant differences in several markers between patients and controls. The receiver operating characteristic curve analysis showed that the areas under the curve for the diagnosis of colorectal polyps using the individual and combined detection of the neutrophil-to-lymphocyte ratio, mean corpuscular haemoglobin (MCH), MCH concentration, cystatin C, triglycerides, low-density lipoprotein cholesterol, carcinoembryonic antigen, *Porphyromonas gingivalis*, *Fusobacterium nucleatum*, *Prevotella intermedia*,* Ruminococcus gnavus*,* Bacteroides ovatus*, and *Parabacteroides distasonis* were 0.696, 0.726, 0.742, 0.771, 0.829, 0.731, 0.785, 0.759, 0.738, 0.786, 0.739, 0.764, 0.757, and 0.996, respectively. Combining 13 markers was better than a single marker regarding the diagnostic effect. Compared to that in normal mucosal tissues, the ratio of positively stained areas for NLRP3, apoptosis-associated speck-like proteins containing a caspase recruitment domain, and interleukin-1β was higher in polyp tissues.

**Conclusion:** Detection of salivary and fecal microbiota, multiple inflammation-related indicators, serum lipid indices, and tumor markers can non-invasively and effectively improve the diagnosis of colorectal polyps.

## Introduction

Colorectal polyps are benign tumours that occur in the rectal mucosa and are commonly observed in digestive tract diseases 1. They can be categorized into three types based on their pathological characteristics: proliferative, inflammatory, and adenomatous. No typical clinical symptoms are observed during the initial stages of polyp development. The serrated pathway and the adenoma-adenocarcinoma pathway are the two main pathways through which colorectal polyps can progress to colorectal cancer (CRC) 2; hence, early diagnosis and prevention of colorectal polyp progression can effectively reduce the incidence of CRC 3. Colonoscopy is the standard clinical examination for polyps 4; however, this invasive procedure can be painful for patients and may lead to bleeding following examination. Currently, several auxiliary screening methods are used in clinical practice, including faecal occult blood, routine blood, and urine routine tests 5. The sensitivity and variability of auxiliary screening methods determine their accuracy 6. Therefore, identifying a convenient, effective, and minimally invasive index for diagnosing and predicting colorectal polyps is essential.

Increasing evidence indicates that the gut microbiota contributes to the carcinogenesis of colorectal polyps, in addition to genetics, age, sex, family history, excessive alcohol consumption, and diets high in animal fats 7, 8. A classification model based on the difference in the intestinal microflora distribution between healthy controls and patients with polyps can distinguish diseases, and the sensitivity of the model can be improved by combining faecal and oral microflora 9, 10. Although pathogenic oral bacteria associated with colorectal polyps were detected in healthy controls, the abundance of this flora was higher in patients with colorectal polyps 10. This shows that the distribution of the oral microflora may lead to susceptibility or resistance to colorectal polyps, which is related to the heterogeneity of colorectal polyps. Previous research by our group identified novel oral and faecal microorganisms as diagnostic indicators for colorectal polyps; however, they were not specific to particular microorganisms 10.

Several studies have shown that inflammation-related indices obtained from routine blood tests, including the platelet-to-lymphocyte ratio (PLR), neutrophil-to-lymphocyte ratio (NLR), systemic immune inflammatory index, and average platelet volume-to-platelet count ratio (MPV/PC), can be used for the diagnosis and prognosis of various malignant tumours, including liver, cervical, endometrial, lung, nasopharyngeal, and oesophageal cancer 11-18. These indicators are widely used in routine examinations for outpatients and inpatients because they are inexpensive and easy to obtain. Most current research has focused on predicting inflammatory factors for CRC, with less focus on predicting colorectal polyps 18-23.

Lipids are indispensable in the process of human metabolism. Abnormal blood lipid metabolism generally manifests as increased plasma total cholesterol and/or triglyceride (TG) levels 24-28. Some studies have also found that patients with colorectal polyps have abnormal changes in high-density lipoprotein cholesterol and low-density lipoprotein cholesterol (LDL-C) 24-26. However, other studies have shown that there is no relationship between blood lipids and colorectal polyps 27,28. Therefore, this specific relationship requires further study.

Studies have reported various cancer types with alterations in glycosylation [19-21. These abnormally expressed glycans and glycoproteins are commonly referred to as tumour-associated glycans/glycolproteins, which can be secreted into the bloodstream and become tumour-related biomarkers 19. Analyses of serum tumour markers, which are convenient and quick, are well accepted by patients and are useful for diagnosing cancer, predicting survival rates, and monitoring recurrence following surgery. Among the available tumour markers, carcinoembryonic antigen (CEA), carbohydrate antigen (CA)19-9, and CA72-4 are widely used for the follow-up of patients with gastrointestinal malignancies 22, 23; however, these methods lack specificity. Furthermore, few studies have reported the relationship between the aforementioned tumour markers and colorectal polyps.

Nod-like receptor protein 3 (NLRP3) is a classic pattern recognition receptor (PRR) that, in addition to apoptosis-associated speck-like proteins containing a caspase recruitment domain (ASC) and pro-caspase-1, constitutes the NLRP3 inflammasome 24. Upon stimulation from both intracellular and extracellular sources, the NLRP3 inflammasome is activated, leading to the conversion of pro-caspase-1 into caspase-1. Caspase-1 then activates the inflammatory factors interleukin (IL)-1β and IL-18 and cleaves gasdermin D, ultimately causing inflammation and pyroptosis 25. Under normal physiological conditions, the NLRP3 inflammasome plays a role in maintaining intestinal environment stability; however, when abnormally activated, it can initiate or promote the development of various intestinal diseases, such as radiation enteritis, inflammatory bowel disease, and CRC 26. The role of NLRP3 in mucosal immunity and colitis is complex, and its relationship with colorectal polyps remains unclear.

In summary, the occurrence of colorectal polyps is a multifactorial chronic process in which dysbiosis of the microbiota, inflammatory responses, and abnormal lipid metabolism play key roles 27. The predictive roles of microorganisms, peripheral blood inflammatory markers, lipid metabolism, and oncological indicators in the prognosis of various tumours have been confirmed in many studies 28-30; however, their application for the early diagnosis of colorectal polyps requires further investigation. This case-control study collected data from patients with colorectal polyps and healthy controls. It analysed the differences in salivary and faecal microbiota, clinical haematological indicators, and inflammasomes in the intestinal tissues between the two groups. This study aimed to explore their value in the early diagnosis of colorectal polyps, with the hope of achieving early detection, diagnosis, and treatment, thereby reducing the cancerization rate.

## Material and Methods

### Participants

In this case-control study, we randomly selected patients diagnosed with colorectal polyps at Shanghai Fifth People's Hospital, Fudan University (Shanghai, China), from 13 May 2022 to 20 November 2023. This research rigorously follows the WHO 2019 classification standards for colorectal tumors 31, with a specific focus on the molecular progression mechanisms of the traditional adenoma-carcinoma pathway. The criteria for inclusion are precisely limited to three types of precancerous adenomatous lesions: tubular adenomas, villous adenomas, and tubulovillous adenomas; the controls were family members of patients with colorectal polyps matched by age, sex, body mass index (BMI), dietary habits, oral hygiene habits, and the absence of intestinal disease by colonoscopy. All participants were older than 18 years. Participants were excluded if they met the following criteria: refused to participate in this study; cognitive impairments that prevented them from cooperating with the researchers; previous history of gastrointestinal disease and family history of colorectal polyps in a first-degree relative; any of the following diseases (autoimmune diseases, such as systemic lupus erythematosus and ankylosing spondylitis; organ failure; cachexia; infectious diseases; or cardiovascular and respiratory diseases); pregnant or lactating; BMI < 18.5 kg/m^2^ or > 32 kg/m^2^; suffering from oral diseases; administered antibiotics, probiotics, microbioactive bacterial preparations, or berberine within the preceding 3 months; and concurrent major disorders or a history of alcohol or drug abuse.

This study was approved by the Ethics Committee of the Shanghai Fifth People's Hospital, Fudan University [(2021) 127] and was conducted in accordance with the World Medical Association Declaration of Helsinki. All enrolled participants provided written informed consent.

## Methods

### Questionnaire survey

Modelled on the 'Oral Health Survey: Basic Methods' (5th edition) published by the World Health Organization (WHO) 32, we designed a questionnaire including the following items: age, sex, education level, occupation, height, weight, smoking habit, oral hygiene behaviour, general health status, and the size, site, number (single and multiple), and pathological type of polyps, among others. The questionnaires were distributed onsite by trained investigators, who provided instructions for completing the forms and collected the completed questionnaires.

### Collection of salivary and fecal samples and methods for detection

Salivary samples were collected from all participants between 8:00 a.m. and 11:00 a.m. Participants were instructed not to eat, drink, smoke, or perform oral hygiene procedures 2 h before sampling. The participants gargled deionised water and collected unstimulated saliva (at least 5 ml) in a plastic cup. If blood was present in the saliva, it was discarded, and the sample was collected again. The collected salivary samples were immediately transferred to a centrifuge tube and centrifuged at 4 ºC for 10 min at 7000 rpm. The supernatant was collected and divided into Eppendorf tubes, which were immediately stored at -80 ºC. Repeated freeze-thawing of salivary samples was avoided during the study.

For all participants, approximately 3-5 g of fresh fecal specimens from the middle section were collected using a special fecal kit (Shanghai Personalbio Technology Co., Ltd, Shanghai, China), immediately frozen at -20 ºC, stored in a dry ice box, transported to the laboratory, and stored at -80 ºC until further analysis.

Salivary and fecal samples were analysed using full-length 16S rRNA sequencing, and the distribution of microbiota in the salivary and faecal samples of patients with colorectal polyps and healthy controls was recorded. Total genomic DNA was isolated from the samples following the protocol provided with the Mag-Bind Blood & Tissue DNA HDQ 96 Kit (M6399-01, Omega, Inc., USA). The concentration and purity of the extracted DNA were assessed using a NanoDrop NC2000 spectrophotometer (Thermo Fisher Scientific, Waltham, Massachusetts, USA) and confirmed by agarose gel electrophoresis. To amplify the nearly complete bacterial 16S rRNA genes, PCR was performed with the forward primer 27F (5'-AGAGTTTGATCMTGGCTCAG-3') and the reverse primer 1492R (5'-ACCTTGTTACGACTT-3'). The DNA was subjected to a two-step PCR amplification process, with the second round incorporating sample-specific 16-bp barcodes into both the forward and reverse primers to enable multiplex sequencing. Each PCR reaction mixture (New England Biolabs, Ipswich, MA, USA) comprised 5 μl of Q5 reaction buffer (5×), 5 μl of Q5 High-Fidelity GC buffer (5×), 0.25 μl of Q5 High-Fidelity DNA Polymerase (5U/μl), 2 μl (2.5 mM) of dNTPs, 1 μl (10μM) of each primer, 2 μl of DNA template, and 8.75 μl of ddH2O. The thermal cycling conditions included an initial denaturation at 98 ºC for 2 min, followed by 25/10 cycles (for the first and second amplification steps, respectively) of denaturation at 98 ºC for 30 s, annealing at 55 ºC for 30 s, and extension at 72 ºC for 90 s, with a final extension of 5 min at 72 ºC. The concentration of the PCR products was determined using the PicoGreen dsDNA Assay Kit (Invitrogen, Carlsbad, CA, USA) after purification with Agencourt AMPure Beads (Beckman Coulter, Indianapolis, IN). The purified amplicons were then quantified individually and pooled in equal proportions for sequencing on the PacBio Sequel platform using Single Molecule Real Time (SMRT) sequencing technology at Shanghai Personal Biotechnology Co., Ltd. (Shanghai, China).

QIIME 2 was employed for microbiome bioinformatics analysis, with slight adjustments to the procedures outlined in the official tutorials (available at https://docs.qiime2.org/2019.4/tutorials/). The process began with the demultiplexing of raw sequence data using the demux plugin, followed by primer trimming with the cutadapt plugin. Subsequently, the Vsearch plugin was utilized for sequence processing, which included merging paired-end reads with fastq_mergepairs, filtering the sequences with fastq_filter, and dereplicating them with derep_fullength. After clustering the unique sequences at 98% similarity using the cluster_size function, chimeric sequences were identified and removed with uchime_denovo. The remaining non-chimeric sequences were then re-clustered at 97% similarity to generate Operational Taxonomic Unit (OTU) representative sequences and an OTU table. The non-singleton OTUs were aligned using mafft and a phylogenetic tree was constructed with fasttree2. Taxonomic classification of the OTU representatives was performed using the RDP Classifier against the Silva database.

### Collection of blood and methods for detection

Blood samples (5 mL) were collected from all participants after 8-12 h of fasting via the cubital vein. All tests were performed at the Clinical Laboratory of Shanghai Fifth People's Hospital, Fudan University. Once the plasma was separated, liver function and blood lipid indicators were analysed using an automatic biochemical analyser (Cobas 8000 c702; Roche Diagnostics GmbH, Mannheim, Germany). Serum tumour biomarker levels were measured using electrochemiluminescence (Modulator E170; Roche Diagnostics, Tokyo, Japan). Liver function and blood lipid indicators included alanine aminotransferase (ALT); total protein; albumin; globulin; albumin/globulin ratio(A/G Ratio); prealbumin; total bilirubin; direct bilirubin; total bile acids; gamma-glutamyl transferase (GGT); alkaline phosphatase (ALP); aspartate aminotransferase (AST); cholinesterase; α-L-Fucosidase; superoxide dismutase (SOD); lactate dehydrogenase (LDH); TG; HDL-C; LDL-C; apolipoprotein A (ApoA); apolipoprotein B (ApoB); lipoprotein(a) [Lp(a)]; apolipoprotein E (ApoE), and Small Dense Low-Density Lipoprotein (sdLDL). Serum tumour biomarker included Carbohydrate Antigen 50 (CA50); Carbohydrate Antigen 242 (CA242); Alpha-Fetoprotein (AFP); Carcinoembryonic Antigen (CEA); Carbohydrate Antigen 19-9 (CA19-9); Carbohydrate Antigen 72-4 (CA72-4); Carbohydrate Antigen 125 (CA125), and Carbohydrate Antigen 15-3 (CA15-3).

### Collection of intestinal polyp and normal intestinal tissue samples and detection of the Nod-like receptor protein 3 inflammasome

Patients with colorectal polyps routinely took polyethylene glycol to clean their intestines before undergoing colonoscopy with an Olympus PCF-Q260AZI (Bond Japan Co.,Ltd; Nishitoriishi, Takaishi, Osaka, Japan), which was inserted into the terminal ileum. During the examination, the location, size, number, and morphology of the polyps were recorded, and images were captured. Biopsy forceps were used to remove the polyp tissue and adjacent normal intestinal mucosa. One part of the tissue was directly fixed with 4% paraformaldehyde for pathological sectioning, while the other part was embedded in CRYOMATRIX (Cryobiomatrix LLC; WILMINGTON, DELAWARE, USA) for the preparation of 6 µm continuous frozen sections. The immunofluorescence experimental procedure was as follows: the sections were removed from the -80 ºC freezer and placed in -20 ºC and 4 ºC freezers for 10 min each. The sections were fixed with pre-cooled acetone at room temperature and washed with phosphate-buffered saline (PBS). The sections were blocked with 10% donkey serum for 1 h. The primary antibody was added and incubated overnight in a refrigerator at 4 ºC. The following day, sections were warmed to room temperature and washed with PBS. Appropriately diluted fluorescent secondary antibodies were added, and the cells were incubated at room temperature for 1 h, followed by washing with PBS. Cell nuclei were stained with DAPI, and a cover slip was applied to the sections. Laser confocal scanning (Eclipse Ni-U; Shanghai, China) was performed using three random fields of view selected from each section for detection and analysis. The green fluorescence-positive area ratio was calculated by dividing the total green fluorescence-positive area by the tissue pixel area.

### Statistical analysis

Data analysis was performed using IBM SPSS Statistics for Windows, version 26.0 statistical software (SPSS Inc. Chicago, IL, USA). Statistical descriptions were performed using frequency/percentage for qualitative data, and intergroup comparisons were analysed using the chi-squared test. The Shapiro-Wilk method was used to test for normality in quantitative data. Quantitative data that conformed to a normal distribution were expressed as the mean ± standard deviation, and intergroup comparisons were analysed using independent sample t-tests. Data that were not normally distributed were expressed as median (interquartile range), [M (Q1,Q3)], and intergroup comparisons were analysed using the non-parametric Mann-Whitney U test. A receiver operating characteristic (ROC) curve was used to test the predictive efficacy of the model, and the area under the curve (AUC) was calculated. An AUC between 0.50 and 0.70 indicated low accuracy, between 0.71 and 0.90 indicated moderate accuracy, and > 0.90 indicated high accuracy. A *P* value < 0.05 was considered significant.

## Results

### Study population

The sample was determined using Power Analysis and Sample Size (PASS) version 12 (NCSS, Kaysville, UT, USA) with a 0.05 significance level and an 80% power based on the results of previous studies 9, 21,27, 28. A total of 106 participants were included in the present study, 47 of whom had colorectal polyps. These included 19 tubular adenomas patients, 13 villous adenomas patients, and 15 tubulovillous adenomas patients. Patients with colorectal polyps and healthy controls were matched for age, sex, BMI, education level, smoking history, frequency of tooth brushing per day, and frequency of oral visits (*P* > 0.05). The demographic data of the participants are presented in Table 1.

### Routine blood test results in patients with colorectal polyps and healthy controls

Routine blood clinical indicators were evaluated using an independent sample t-test for pairwise comparisons between patients with colorectal polyps and healthy controls. Table 2 presents the results of the study. Compared with healthy controls, patients with colorectal polyps showed increased levels of neutrophils, NLR, mean haemoglobin volume, and mean haemoglobin concentration, whereas lymphocyte count, red blood cell count, and haemoglobin level were decreased (*P* < 0.05).

### Liver function/blood lipid results in patients with colorectal polyps and healthy controls

Liver function and blood lipid indicators were analysed using an independent sample t-test in pairwise comparisons of patients with colorectal polyps and controls. Table 3 presents the results of the study. Cystatin C, TG, and LDL-C concentrations were higher (*P* < 0.001) in patients with colorectal polyps than in controls. Compared to that in healthy controls, total cholesterol in patients with colorectal polyps increased, but the difference was not significant (*P* = 0.073).

### Serum tumor marker results in patients with colorectal polyps and healthy controls

Levels of carbohydrate antigen 50 (CA50) and CEA in patients with colorectal polyps were significantly higher than in controls (*P* = 0.011 and *P* < 0.001, respectively) in Table 4.

### Saliva and fecal microbiota results in patients with colorectal polyps and healthy controls

**Figure 1:**
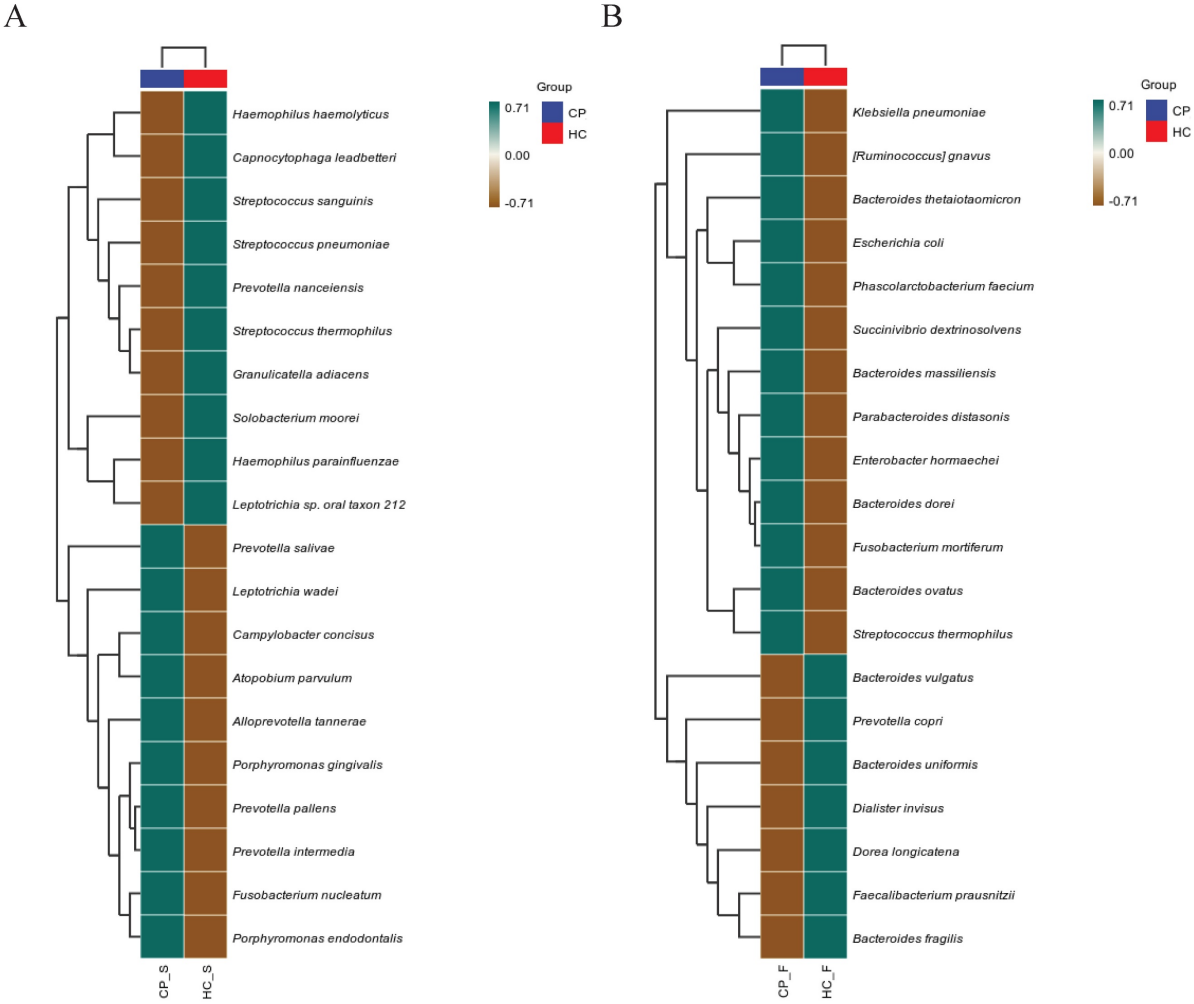
**Heatplots of correlation between OTUs detected in (A) salivary and (B) fecal samples of patients with colorectal polyps and healthy controls. Abbreviations:** CP_S: salivary samples of colorectal polyp patients; CP_F: fecal samples of colorectal polyp patients; HC_S: salivary samples of healthy controls; HC_F: fecal samples of healthy controls.

**Figure 2:**
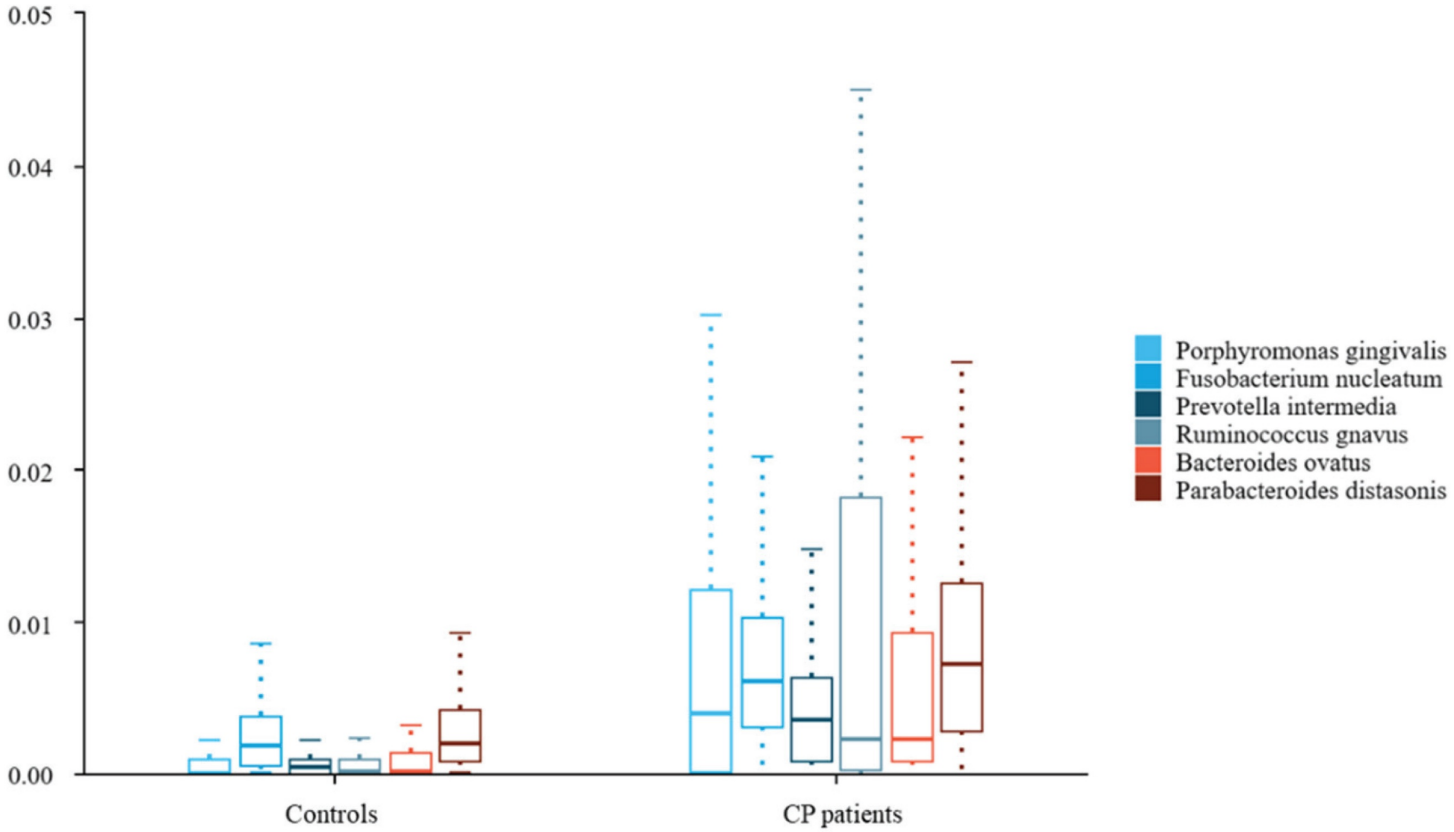
** Differences in saliva and fecal microbiota between colorectal polyps patients and healthy controls. Abbreviations:** CP patients: colorectal polyps patients

To further compare microbiota differences, we constructed heatplots using data of the top 20 at the species levels (Figure 1A and B). The abundance of *Porphyromonas gingivalis*, *Fusobacterium nucleatum*, and *Prevotella intermedia* in the saliva of patients with colorectal polyps was higher than that in healthy controls. Additionally, the abundance of *Ruminococcus gnavus*, *Bacteroides ovatus*, and *Parabacteroides distasonis* in the faeces was significantly increased (all *P* < 0.05), as shown in Figure 2.

### Expression of Nod-like receptor protein 3, apoptosis-associated speck-like proteins containing a caspase recruitment domain, and interleukin-1β in intestinal polyp tissues and normal intestinal mucosa

**Figure 3:**
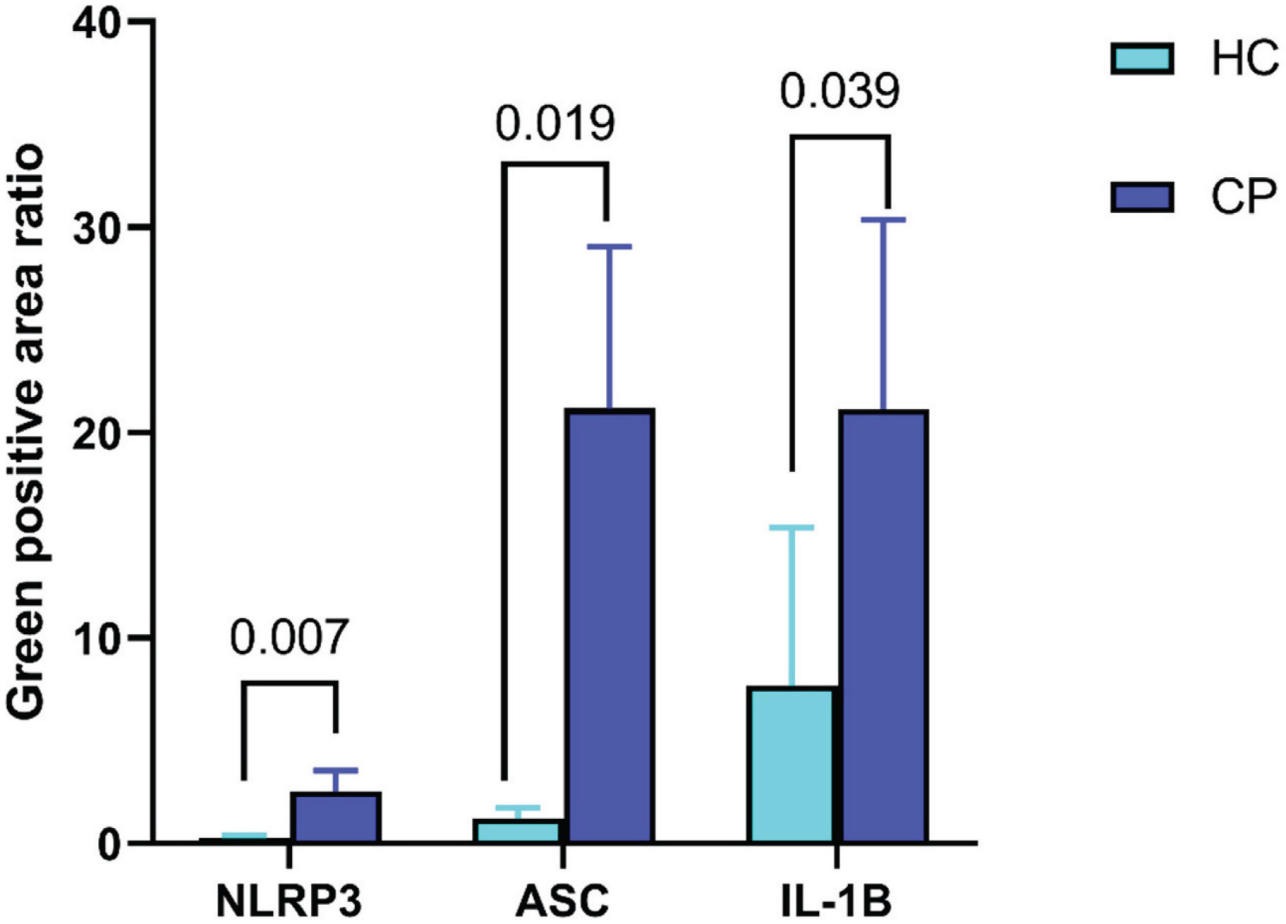
** Expression of NLRP3, ASC, IL-1β in intestinal polyp tissues and normal intestinal mucosa. Abbreviations**: HC: healthy controls; CP: colorectal polyps patients.

Compared to that in normal mucosal tissues, the ratio of positively stained areas for NLRP3, ASC, and IL-1β increased in polyp tissues (*P* < 0.05) as shown in Figure 3.

### Diagnostic value of salivary and fecal microbiota, blood routine tests, blood lipid levels, and tumor inflammation marker levels for colorectal polyps

**Figure 4:**
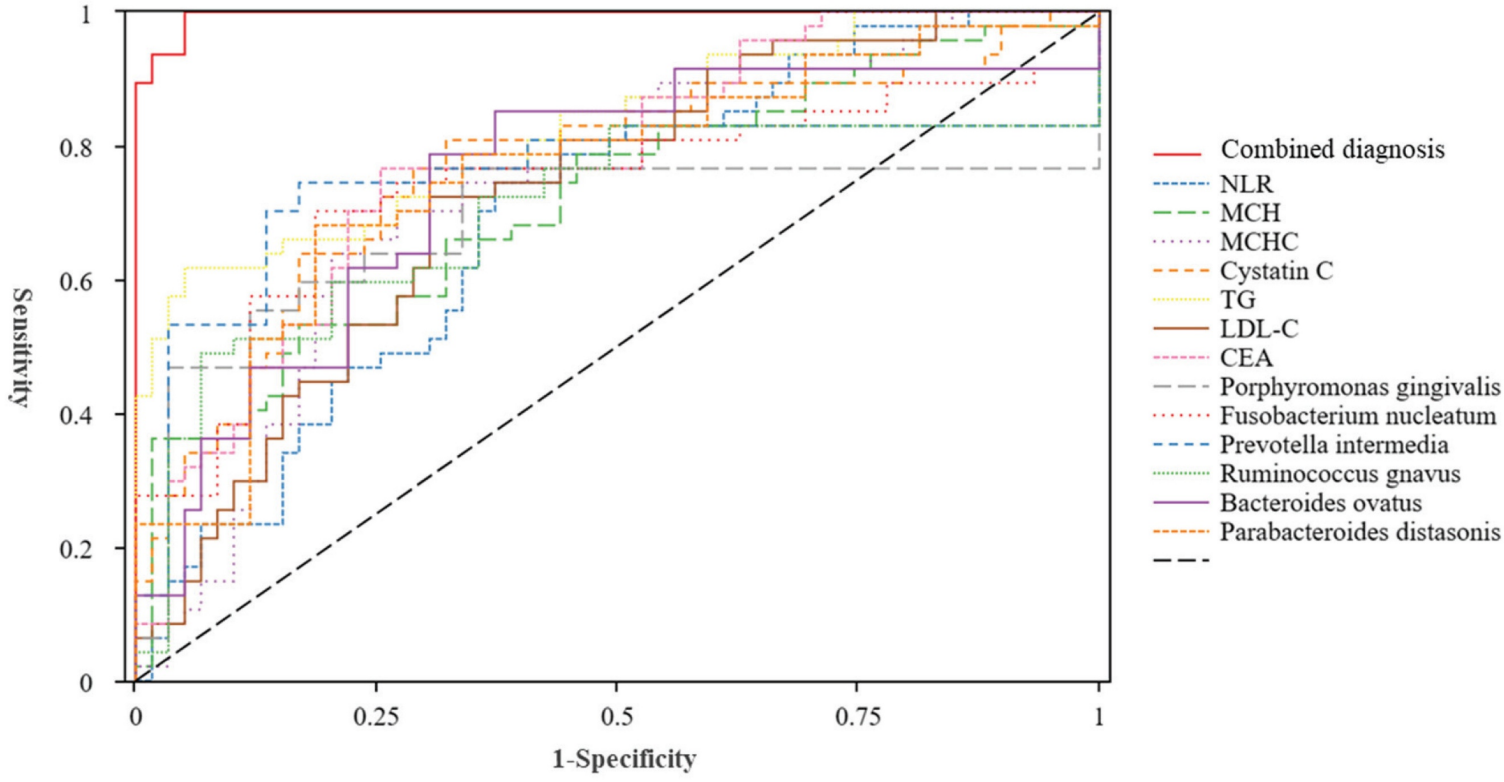
** ROC curve of saliva and fecal microbiota, blood routine, blood lipids, and tumor inflammatory indicators in the diagnosis of colorectal polyps. Abbreviations**: NLR: Neutrophil-Lymphocyte Ratio; MCH: Mean Corpuscular Hemoglobin; MCHC: Mean Corpuscular Hemoglobin Concentration; TG: Triglycerides; LDL-C: Low-Density Lipoprotein Cholesterol; CEA: Carcinoembryonic Antigen.

To explore the diagnostic value of the NLR, mean corpuscular haemoglobin (MCH), MCH concentration (MCHC), cystatin C, TG, LDL-C, CEA, *P. gingivalis*, *F. nucleatum*, *P. intermedia*, *R. gnavus*, *B. ovatus*, and *P. distasonis* in colorectal polyps, we plotted ROC curves. The combined diagnosis of the indicators had an AUC value of 0.996. Figure 4 and Table 5 showed that each indicator has a comparable diagnostic value for colorectal polyps. The AUC value of the combined diagnosis of NLR, MCH, MCHC, cystatin C, TG, LDL-C, and CEA was 0.976, with a sensitivity of 87.2% and a specificity of 99.6%. Furthermore, the diagnostic value of *P. gingivalis*, *F. nucleatum*, *P. intermedia*, *R. gnavus*, *B. ovatus*, and *P. distasonis* for colorectal polyps was 0.868, with a sensitivity of 89.4% and specificity of 71.2%.

## Discussion

CRC develops through two major molecular pathways: the classic adenoma-adenocarcinoma sequence (~70% of cases) and the serrated neoplasia pathway (SNP, ~30%), characterized by hypermethylation of CpG islands and KRAS mutations 33. While the adenoma pathway is well-characterized in microbiome studies, the role of microbial dysbiosis in the SNP remains poorly understood 2, 34. This study focuses on the adenomatous polyp spectrum, and future investigations integrating serrated polyps will be essential to comprehensively evaluate microbial contributions to CRC pathogenesis.

Although the predictive roles of microorganisms, peripheral blood inflammatory markers, lipid metabolism, and oncological indicators in the prognosis of various tumours have been confirmed in many studies, their application for the early diagnosis of colorectal polyps requires further investigation. This case-control study collected data from patients with colorectal polyps and healthy controls. We analysed the differences in salivary and fecal microbiota, clinical haematological indicators, and inflammasomes in the intestinal tissues between the two groups. The ROC showed that the AUC for the diagnosis of colorectal polyps using the individual and combined detection of the NLR, MCH, MCHC, cystatin C, TG, LDL-C, CEA, *P. gingivalis, F. nucleatum, P. intermedia, R. gnavus, B. ovatus, and P. distasonis* were 0.696, 0.726, 0.742, 0.771, 0.829, 0.731, 0.785, 0.759, 0.738, 0.786, 0.739, 0.764, 0.757, and 0.996, respectively. Therefore, by combining these biomarkers, a more comprehensive and accurate diagnosis of colorectal polyps could be achieved, thereby providing a reference for clinical diagnosis and treatment.

According to the 2022 GLOBOCAN data, CRC is the third most commonly diagnosed cancer globally and the second leading cause of cancer-related deaths despite the introduction of improved early detection screening and advancements in treatment 35. Over 90% of CRC cases are adenocarcinomas, which arise as malignant growths in the glandular epithelial cells of the large intestine, encompassing both the colon and rectum 36. The focus of this study was the alignment of colorectal polyps with adenoma-carcinoma sequences. Previous studies showed that approximately 90% of colorectal polyps occur in individuals aged 50 years and older, with significantly more male patients than females 3, 37. The age and sex distribution characteristics of the patients with colorectal polyps in our study were consistent with these findings.

The link between inflammation and malignancy has been well-established since it was first proposed in 1863 38. Inflammation causes systemic changes in the tumour microenvironment, which facilitates tumour progression. Neutrophils regulate the tumour microenvironment and produce cytokines that promote angiogenesis and tumour cell proliferation and migration 39. Lymphocytes play an important role in antitumour immunity by promoting tumour cell apoptosis and inhibiting tumour cell progression. Studies have shown that a high NLR is associated with an increase in colorectal polyps 40, 41. Chronic inflammation damages normal colorectal epithelial cells by releasing multiple inflammatory mediators that damage endothelial cells and enhance vascular permeability. Studies have shown that the NLR can be used as a risk assessment indicator for the malignant transformation of adenomatous polyps 42-44. The results of this study are consistent with these findings. The NLR can be influenced by various factors, such as infection, autoimmune diseases, and medication treatment, which may interfere with the determination of colorectal polyps using the NLR. Additionally, there may be differences in the NLR cutoff values among different studies 40, 43, 44, which also poses certain difficulties for clinical application. Further large-sample multicentre studies are needed to determine more accurate NLR cutoff values and assessment criteria.

Some studies have indicated that serum TG and cholesterol levels are associated with an increased risk of colorectal polyps 45-48. In contrast, other studies have either failed to confirm such a link or have suggested an inverse relationship between serum lipid levels and colorectal polyps 49, 50. A review of 37 articles showed that the levels of TG, total cholesterol, and LDL-C in patients with colorectal polyps were higher than those in controls 51, consistent with our findings. Although the underlying mechanisms have not been fully elucidated, two pathways may be involved 46. One pathway suggests that lipid abnormalities are involved in the development of hyperinsulinaemia and insulin resistance. Through interactions with the IGF-1 receptor, they inhibit apoptosis, promote the proliferation of large bowel cells, and induce carcinogenesis. Second, lipid abnormalities may be associated with bile acid production, increasing CRC risk. Therefore, it is reasonable to expect that serum lipids play a role in polyp recurrence.

Serum tumour markers are important auxiliary examination tools for the clinical diagnosis and prognostic evaluation of tumours 22, 29. CEA is one of the most widely used tumour markers. It is a specific acidic glycoprotein of human embryonic antigen. Previous studies found that the CEA level in patients with colorectal polyps was higher than that in healthy controls 22, 29, 52, consistent with the results of this study. We confirmed that the diagnostic value of a single CEA index was better than that of several other tumour markers.

Studies have shown that the abundance of microbiota, such as *Streptococcus*, *Prevotella*, *P. gingivalis,* and *F. nucleatum,* in the oral cavity of patients with colorectal polyps is significantly higher than that in controls 10, 53, consistent with our study results. These oral pathogens can not only directly invade colorectal tumours, but may also affect routine blood indicators by triggering inflammatory responses.* P. gingivalis* can produce extracellular enzymes, such as gingipains, which can degrade the extracellular matrix of the intestinal mucosa, disrupt the barrier function of the intestinal mucosa, and make it more susceptible to invasion by harmful substances, thereby promoting the occurrence of colorectal polyps. Our previous study found that the diagnostic performance of the oral microbiota for colorectal polyps was significant; however, it did not target a specific microbiota 10. This study further investigated three periodontal pathogens and found that their diagnostic performance for colorectal polyps was 75.9%, 73.8%, and 78.6%, indicating a high diagnostic value.

Conde-Pérez, K et al. 54 demonstrated a significant over-representation of *Parvimonas*, *Fusobacterium*, and *Bacteroides fragilis* in the stool samples of CRC patients and further proposed that a consortium comprising *Fusobacterium*, *Parvimonas*, *Bacteroides*, and *Faecalibacterium* could be harnessed to develop a highly effective non-invasive fecal test for the early detection of CRC. Datorre, J G et al. 55 also indicated the potential of fecal *F. nucleatum* detection as a non-invasive biomarker for colorectal cancer screening. However, our findings revealed a significant increase in the abundance of *R. gnavus*, *B. ovatus*, and *P. distasonis* in the feces, which deviates from the aforementioned observations. The discrepancies among the studies may primarily be attributed to differences in the study subjects. Firstly, their studies focused on CRC patients, whereas our study centered on colorectal polyps. Secondly, the methodologies employed for detection varied; the ultrasensitive ddPCR technique utilized by Datorre et al. exhibits superior sensitivity compared to 16S rRNA sequencing, and their participants were FIT-positive individuals. Thirdly, regional variations in diet and lifestyle habits can significantly influence the composition and distribution of the gut microbiota.

Remarkably, Raúl Y Tito 56 well-established microbiome CRC targets, such as *F. nucleatum*, did not significantly associate with CRC diagnostic groups (healthy, adenoma and carcinoma) when controlling primary microbial covariates about transit time, fecal calprotectin (intestinal inflammation), body mass index and so on. Their research highlights the importance of controlling covariates in CRC microbiome studies, reevaluating existing associations, and exploring the complex relationship between the gut microbiome and disease. This will provide important guidance for future research and advance the development of CRC microbiome in clinical applications. Our study controlled for confounding factors such as age, sex, BMI, education level, diabetes, hypertension, smoking, and alcohol consumption, but not transit time, fecal calprotectin (intestinal inflammation). Moving forward, the implementation of standardized methodologies, cross-population validation frameworks, and comprehensive covariate adjustment strategies will be paramount to propel advancements in CRC microbiome research.

*R. gnavus* has been linked to a range of intestinal and extraintestinal conditions and is consistently overrepresented in individuals with inflammatory bowel disease and metabolic disorders. Research suggests that *R. gnavus* may play a role in the gut-brain and gut-liver axes. Nevertheless, it remains unclear whether *R. gnavus* actively contributes to the onset of these diseases (acting as a causative agent) or simply thrives because of disease-induced alterations in the microbial environment and the physiological state of the host, which could promote its proliferation in the gut 57. Our study found that the diagnostic value of *R. gnavus* for colorectal polyps was 73.9%, with a sensitivity of 48.9% and specificity of 93.2%, a finding that has not been previously reported in related studies.

The relationship between* B. ovatus* and human health is complex, as it can be beneficial and potentially pathogenic. The beneficial effects mainly include breaking down complex carbohydrates and proteins and producing nutrients, which provide energy for the host and promote the absorption of nutrients in the gut, thus maintaining the balance of the gut microbiota 58. These harmful effects mainly involve opportunistic infections that potentially breach the intestinal mucosal barrier, enter the bloodstream or other tissues and organs, and cause opportunistic infections, such as sepsis, abdominal infections, and pelvic infections 59, 60. Our study found that the abundance of *B. ovatus* in the faeces of patients with colorectal polyps increased. It is speculated that this may be due to dysbiosis of the gut microbiota, leading to an increase in gut immune-inflammatory responses, which in turn triggers systemic inflammatory responses; however, the specific causal relationships require further research.

*P. distasonis* may offer protective benefits against several illnesses, such as type II diabetes, CRC, and inflammatory bowel disease 61. Some studies have proposed that this bacterium could be used as a probiotic to enhance human digestive health 62. Nonetheless, other experimental evidence presents conflicting findings, indicating pathogenic effects in different disease models 63, 64. This implies that the role of *P. distasonis* may be dual, depending on the specific circumstances. *P. distasonis* and its related metabolites may serve as biomarkers for disease diagnosis and provide a basis for early diagnosis and precision medicine.

The NLRP3 inflammasome is widely present in various cell types. It can induce the production of pro-inflammatory factors, such as IL-10 and IL-18, through the activation of the NF-κB pathway and stimulation of multiple signalling molecules. In addition, it can disrupt cancer cell membranes, triggering cancer cell pyroptosis 24. Animal studies have explored the role of the NLRP3 inflammasome in CRC development. For example, Son et al. found that the levels of the NLRP3 inflammasome and myeloperoxidase in colon tissues gradually increased during the process of inducing a CRC mouse model with azoxymethane and dextran sulphate sodium 65. Studies have suggested that the NLRP3 inflammasome may be linked to the low-grade chronic inflammation associated with obesity and the development of CRC 66-68. Our results showed that, compared to that in normal mucosal tissues, the ratio of positively stained areas for NLRP3, ASC, and IL-1β increased in polyp tissues. This finding has not been previously reported in related studies, and the specific relationships and mechanisms require further research and exploration.

Our study was designed as a single-centre, cross-sectional study focusing on the diagnostic accuracy of adenomatous polyps, with an explicit exclusion of non-adenomatous polyps. The sample consisted predominantly of middle-aged and elderly inpatients from a single hospital, which may have introduced a selection bias. Due to resource constraints and the exploratory nature of the study, we did not collect follow-up data. Additionally, the study lacked metagenomics and metabolomics to explore microbiota function, analysed bacterial metabolites and host interplay, and without FMT or organoid co-culture, it can't verify target strains' causal role in pathology, impeding clinical application. Further large-scale, prospective cohort clinical studies and long-term follow-up data on patient prognosis and treatment are needed to overcome these limitations and to provide more convenient and rapid screening indicators for the timely detection of colorectal polyps. Concurrently, an in-depth investigation will be conducted into the underlying mechanisms through which oral pathogens contribute to the initiation and progression of colorectal polyps. This will involve a comprehensive exploration of the molecular and cellular pathways by which these pathogens interact with the host environment, potentially leading to the development of colorectal polyps.

## Conclusion

In conclusion, salivary and fecal microbiota, routine blood tests, blood lipids, and serum tumour markers are commonly used biomarkers for screening and diagnosing colorectal polyps. Each of these biomarkers has different levels of sensitivity and specificity, and may have limitations when used individually; however, when used in combination, they can enhance the accuracy and reliability of diagnoses. Different biomarkers may reflect the presence or progression of colorectal polyps from different perspectives, suggesting potential complementarity. By combining these biomarkers, a more comprehensive and accurate diagnosis of colorectal polyps could be achieved, thereby providing a reference for clinical diagnosis and treatment. This approach can avoid unnecessary colonoscopies and increase the acceptance rate of population screening, thereby improving the effectiveness of CRC screening and reducing related medical burdens and costs. In the future, it will be necessary to develop non-invasive diagnostic technologies for colorectal polyps and CRC based on microbiota and blood indicators, which will benefit cancer prevention and control, and public health.

## Figures and Tables

**Table 1:** Demographic characteristics of the subjects

Characteristics		HC (n=59)	CP (n = 47)	*P*-value
Age (mean ± SD)		61.44±6.78	60.27±9.47	0.463
BMI (mean ± SD) kg/m2		25.68±3.07	25.54±3.08	0.819
Sex				0.528
	Male	38(64.6)	33(70.2)	
	Female	21(35.6)	14(29.8)	
Educations level				0.985
	Illiteracy	2(3.4)	2(4.3)	
	Junior school	15(25.4)	13(27.7)	
	Junior high school	31(52.5))	24(51.1)	
	High school or above	11(18.6)	8(17.0)	
Vocation				0.894
	Retiree	32(54.2)	25(53.2)	
	Farmer	5(8.5)	3(6.4)	
	Worker	22(37.2)	19(40.4)	
Diabetes				0.298
	Yes	7(11.9)	9(19.1)	
	No	52(88.1)	38(80.9)	
Hypertension				0.867
	Yes	13(22.0)	11(23.4)	
	No	46(78.0)	36(76.6)	
Smoking status				0.286
	Never	49(83.1)	33(70.2)	
	Ex	4(6.8)	5(10.6)	
	Current	6(10.2)	9(19.1)	
Alcohol consumption				0.720
	Never	41(69.5)	33(70.2)	
	Ex	3(5.1)	4(8.5)	
	Current	15(25.4)	10(21.3)	
Meat-eating frequency				0.829
	1-2 times/week	20(33.9)	15(31.9)	
	>2 times/week	39(66.1)	32(68.1)	
Defecation frequency				0.737
	1-2 times/week	4(6.8)	4(8.5)	
	1-2 times/day	55(93.2)	43(91.5)	
DMFT, M (IQR)		7.85±2.68	7.60±2.53	0.624
History of periodontitis				0.210
	Yes	42(71.2)	28(59.6)	
	No	17(28.8)	19(40.4)	
Frequency of toothbrushing				0.745
	<2 times/day	32(54.2)	24(51.1)	
	≥2 times/day	27(45.8)	23(48.9)	
Frequency of tooth flossing				0.805
	Not every day	50(84.7)	39(83.0)	
	Every day	9(15.3)	8(17.0)	
Frequency of dental visits				0.319
	≤1 time/year	27(45.8)	17(36.2)	
	> 1 time/year	32(54.2)	30(63.8)	
Exercise				0.924
	Never	26(44.1)	22(46.8)	
	Occasionally	21(35.6)	15(31.9)	
	Frequently	12(20.3)	10(21.3)	
Polyp position				
	Rectum	12(25.5)	-	
	Sigmoid colon	15(31.9)	-	
	Descending colon	8(17.0)	-	
	Transverse colon	9(19.1)	-	
	Ascending colon	3(6.4)	-	
Polyp number				
	Single	16(34.0)	-	
	More than 2	31(66.0)	-	
Polyp size (cm)		1.14±0.39		

**Abbreviations:** HC: healthy controls; CP: colorectal polyps patients; P: significance of differences between healthy controls and patients with colorectal polyps.

**Table 2:** Comparison of blood routine clinical indicators between colorectal polyps patients and healthy controls

	HC	CP	*P*
WBC	5.92±1.34	5.92±1.73	0.490
Neutrophil Percentage	56.11±8.94	61.06±6.00	0.004
Lymphocyte Percentage	34.24±8.66	29.76±5.29	0.003
Monocyte Percentage	8.81±11.44	6.88±1.26	0.256
Neutrophils	3.33±0.92	12.69±59.08	0.002
Lymphocytes	2.03±0.68	1.74±0.52	0.034
Monocytes	0.44±0.14	0.41±0.14	0.224
RBC	4.96±0.43	4.55±0.59	<0.001
Hb	147.07±17.15	140.89±18.32	0.038
MCH	29.59±1.95	31.02±1.98	<0.001
MCHC	330.51±13.29	340.64±10.03	<0.001
Platelets	239.92±80.11	221.43±69.60	0.251
PDW	11.95±2.46	12.43±2.79	0.714
MPV	10.33±1.16	10.49±1.18	0.470
NLR	1.84±0.91	2.43±1.01	0.001
PLR	133.00±91.39	136.45±52.88	0.121
LMR	5.01±1.98	4.41±1.16	0.106
RLR	7.30±3.33	8.08±3.04	0.058
SII	451.31±386.21	466.61±172.96	0.055
PIV	197.35±154.93	193.47±97.74	0.459
MPV/PLT	0.05±0.02	0.054±0.02	0.233
PDW/PLT	0.06±0.02	0.06±0.03	0.198

**Abbreviations:** HC: healthy controls; CP: colorectal polyps patients; WBC: White Blood Cell; RBC: Red Blood Cells; Hb: Hemoglobin; MCH: Mean Corpuscular Hemoglobin; MCHC: Mean Corpuscular Hemoglobin Concentration; PDW: Platelet Volume Distribution Width; MPV: Mean Platelet Volume; NLR: Neutrophil-Lymphocyte Ratio; PLR: Platelet-Lymphocyte Ratio; LMR: Lymphocyte-Monocyte Ratio; RLR: Red Blood Cell Distribution Width to Lymphocyte Ratio; SII: Systemic Inflammatory Index; PIV: Plateletcrit; MPV/PLT: Mean Platelet Volume/ Platelet Count; PDW/PLT: Platelet Volume Distribution Width/ Platelet Count.

**Table 3:** Comparison of liver function/blood lipid indicators between colorectal polyps patients and healthy controls

	HC	CP	*P*
ALT	24.98±15.63	21.4±13.35	0.214
Total Protein	70.48±4.61	69.71±4.58	0.395
Albumin	46.31±5.69	45.46±5.03	0.424
Globulin	26.22±3.41	24.87±4.97	0.099
A/G Ratio	1.86±0.44	1.87±0.33	0.945
Prealbumin	1.71±7.30	0.26±0.05	0.177
Total Bilirubin	14.12±6.98	12.28±6.12	0.157
Direct Bilirubin	5.04±1.85	4.67±2.12	0.338
Total Bile Acids	3.82±.192	4.09±2.93	0.593
GGT	29.90±31.75	38.04±53.21	0.330
ALP	69.75±16.21	82.24±58.12	0.118
AST	19.34±5.52	22.37±11.46	0.076
Cholinesterase	9301.61±2173.68	8604.51±1962.97	0.090
α-L-Fucosidase	22.90±5.57	21.90±5.57	0.344
SOD	173.08±17.06	170.89±19.54	0.539
LDH	171.27±35.27	170.77±23.71	0.933
Urea	6.47±5.55	5.38±1.52	0.194
Creatinine	74.00±16.56	76.04±22.95	0.596
Cystatin C	0.78±0.13	0.98±0.28	< 0.001
Total Cholesterol	4.03±1.65	4.52±0.99	0.073
TG	1.36±0.40	2.278±0.93	< 0.001
HDL-C	1.23±0.36	1.27±0.37	0.579
LDL-C	2.55±0.0.69	3.12±0.64	< 0.001
ApoA	1.94±1.02	2.08±0.0.88	0.469
ApoB	0.92±0.20	0.95±0.23	0.588
Lp(a)	35.75±52.32	44.16±50.94	0.408
ApoE	4.48±1.37	4.28±1.19	0.423
sdLDL	1.03±0.39	0.93±0.47	0.228

**Abbreviations:** HC: healthy controls; CP: colorectal polyps patients; ALT: Alanine aminotransferase; A/G Ratio: Albumin/Globulin Ratio; GGT: Gamma-Glutamyl Transferase; ALP: Alkaline Phosphatase; AST: Aspartate Aminotransferase; SOD: Superoxide Dismutase; LDH: Lactate Dehydrogenase; TG: Triglycerides; HDL-C: High-Density Lipoprotein Cholesterol; LDL-C: Low-Density Lipoprotein Cholesterol; ApoA: Apolipoprotein A; ApoB: Apolipoprotein B; Lp(a): Lipoprotein(a); ApoE: Apolipoprotein E; sdLDL: Small Dense Low-Density Lipoprotein.

**Table 4:** Comparison of Serum tumor markers between colorectal polyps patients and healthy controls

	HC	CP	*P*
CA50	6.47±4.51	15.72±26.91	0.011
CA242	14.00±18.11	18.21±29.72	0.371
AFP	3.12±1.72	3.36±1.80	0.623
CEA	1.46±0.86	2.75±2.03	< 0.001
CA19-9	8.56±5.41	18.12±67.97	0.284
CA72-4	3.23±3.81	3.82±4.03	0.444
CA125	16.64±12.59	14.79±11.59	0.438
CA15-3	9.20±3.84	8.33±3.89	0.252

**Abbreviations:** CA50: Carbohydrate Antigen 50; CA242: Carbohydrate Antigen 242; AFP: Alpha-Fetoprotein; CEA: Carcinoembryonic Antigen; CA19-9: Carbohydrate Antigen 19-9; CA72-4: Carbohydrate Antigen 72-4; CA125: Carbohydrate Antigen 125; CA15-3: Carbohydrate Antigen 15-3.

**Table 5:** Diagnostic value of saliva and fecal microbiota, blood routine, blood lipids, and tumor inflammatory indicators in colorectal polyp.

	AUC	95% CI	*P*	Sensitivity	Specificity	Cut-off
Combined diagnosis	0.996	0.990 ~ 1.002	< 0.001	1.000	0.949	0.149
NLR	0.696	0.597 ~ 0.796	0.001	0.745	0.627	1.888
MCH	0.726	0.628 ~ 0.823	< 0.001	0.532	0.831	31
MCHC	0.742	0.646 ~ 0.837	< 0.001	0.638	0.797	339
Cystatin C	0.771	0.678 ~ 0.865	< 0.001	0.809	0.678	0.8
TG	0.829	0.751 ~ 0.908	< 0.001	0.617	0.949	1.98
LDL-C	0.731	0.636 ~ 0.826	< 0.001	0.723	0.695	2.82
CEA	0.785	0.699 ~ 0.871	< 0.001	0.766	0.746	1.7
Porphyromonas gingivalis	0.759	0.664 ~ 0.854	< 0.001	0.553	0.881	0.003
Fusobacterium nucleatum	0.738	0.634 ~ 0.841	< 0.001	0.702	0.814	0.004
Prevotella intermedia	0.786	0.692 ~ 0.880	< 0.001	0.745	0.831	0.001
Ruminococcus gnavus	0.739	0.642 ~ 0.836	< 0.001	0.489	0.932	0.004
Bacteroides ovatus	0.764	0.673 ~ 0.855	< 0.001	0.787	0.695	0.001
Parabacteroides distasonis	0.757	0.663 ~ 0.851	< 0.001	0.681	0.814	0.004

**Abbreviations:** NLR: Neutrophil-Lymphocyte Ratio; MCH: Mean Corpuscular Hemoglobin; MCHC: Mean Corpuscular Hemoglobin Concentration; TG: Triglycerides; LDL-C: Low-Density Lipoprotein Cholesterol; CEA: Carcinoembryonic Antigen.

## Abbreviation

CRC: colorectal cancer
NLRP3: Nod-like receptor protein 3
ASC: apoptosis-associated speck-like proteins containing a caspase recruitment domain
IL-1β: inflammatory factors interleukin (IL)-1β
WBC: White Blood Cell
RBC: Red Blood Cells
Hb: Hemoglobin
MCH: Mean Corpuscular Hemoglobin
MCHC: Mean Corpuscular Hemoglobin Concentration
PDW: Platelet Volume Distribution Width
MPV: Mean Platelet Volume
NLR: Neutrophil-Lymphocyte Ratio
PLR: Platelet-Lymphocyte Ratio
LMR: Lymphocyte-Monocyte Ratio
RLR: Red Blood Cell Distribution Width to Lymphocyte Ratio
SII: Systemic Inflammatory Index
PIV: Plateletcrit
MPV/PLT: Mean Platelet Volume/ Platelet Count
PDW/PLT: Platelet Volume Distribution Width/ Platelet Count
ALT: Alanine aminotransferase
A/G Ratio: Albumin/Globulin Ratio
GGT: Gamma-Glutamyl Transferase
ALP: Alkaline Phosphatase
AST: Aspartate Aminotransferase
SOD: Superoxide Dismutase
LDH: Lactate Dehydrogenase
TG: Triglycerides
HDL-C: High-Density Lipoprotein Cholesterol
LDL-C: Low-Density Lipoprotein Cholesterol
ApoA: Apolipoprotein A
ApoB: Apolipoprotein B
Lp(a): Lipoprotein(a)
ApoE: Apolipoprotein E
sdLDL: Small Dense Low-Density Lipoprotein
CA50: Carbohydrate Antigen 50
CA242: Carbohydrate Antigen 242
AFP: Alpha-Fetoprotein
CEA: Carcinoembryonic Antigen
CA19-9: Carbohydrate Antigen 19-9
CA72-4: Carbohydrate Antigen 72-4
CA125: Carbohydrate Antigen 125
CA15-3: Carbohydrate Antigen 15-3

## References

[1] Monreal-Robles R, Jáquez-Quintana JO, Benavides-Salgado DE, González-González JA (2021). Serrated polyps of the colon and rectum: a concise review. Rev Gastroenterol Mex (Engl Ed).

[2] Nguyen LH, Goel A, Chung DC (2020). Pathways of Colorectal Carcinogenesis. Gastroenterology.

[3] Sninsky JA, Shore BM, Lupu GV, Crockett SD (2022). Risk Factors for Colorectal Polyps and Cancer. Gastrointest Endosc Clin N Am.

[4] Mareth K, Gurm H, Madhoun MF (2022). Endoscopic Recognition and Classification of Colorectal Polyps. Gastrointest Endosc Clin N Am.

[5] Jayasinghe M, Prathiraja O, Caldera D (2023). Colon Cancer Screening Methods: 2023 Update. Cureus.

[6] Jain S, Maque J, Galoosian A, Osuna-Garcia A, May FP (2022). Optimal Strategies for Colorectal Cancer Screening. Curr Treat Options Oncol.

[7] Pop OL, Vodnar DC, Diaconeasa Z (2020). An Overview of Gut Microbiota and Colon Diseases with a Focus on Adenomatous Colon Polyps. Int J Mol Sci.

[8] Mangifesta M, Mancabelli L, Milani C (2018). Mucosal microbiota of intestinal polyps reveals putative biomarkers of colorectal cancer. Sci Rep.

[9] Russo E, Gloria LD, Nannini G (2023). From adenoma to CRC stages: the oral-gut microbiome axis as a source of potential microbial and metabolic biomarkers of malignancy. Neoplasia.

[10] Zhang L, Feng Z, Li Y (2023). Salivary and fecal microbiota: potential new biomarkers for early screening of colorectal polyps. Front Microbiol.

[11] Morelli D, Cantarutti A, Valsecchi C (2023). Routine perioperative blood tests predict survival of resectable lung cancer. Sci Rep.

[12] Huang B, Wu FC, Wang WD (2023). The prognosis of breast cancer patients with bone metastasis could be potentially estimated based on blood routine test and biochemical examination at admission. Ann Med.

[13] Zhou Y, Walter FM, Mounce L (2022). Identifying opportunities for timely diagnosis of bladder and renal cancer via abnormal blood tests: a longitudinal linked data study. Br J Gen Pract.

[14] Cao W, Shao Y, Wang N, Jiang Z, Yu S, Wang J (2022). Pretreatment red blood cell distribution width may be a potential biomarker of prognosis in urologic cancer: a systematic review and meta-analysis. Biomark Med.

[15] Yamamoto T, Kawada K, Obama K (2021). Inflammation-Related Biomarkers for the Prediction of Prognosis in Colorectal Cancer Patients. Int J Mol Sci.

[16] Vuković A, Kuna K, Lončar BB (2021). THE ROLE OF SALIVARY AND SERUM CA125 AND ROUTINE BLOOD TESTS IN PATIENTS WITH OVARIAN MALIGNANCIES. Acta Clin Croat.

[17] Lennon AM, Buchanan AH, Kinde I (2020). Feasibility of blood testing combined with PET-CT to screen for cancer and guide intervention. Science.

[18] Merrill AY, Garland MM, Howard-McNatt M, Isnassuos M, Perry KC, Levine EA (2019). What Is the Utility of Routine Complete Blood Count, Liver Function Tests, and Chest X-ray in the Evaluation of Patients with Clinically Node-Negative Breast Cancer?. Am Surg.

[19] He M, Zhou X, Wang X (2024). Glycosylation: mechanisms, biological functions and clinical implications. Signal Transduct Target Ther.

[20] Wang M, Zhu J, Lubman DM, Gao C (2019). Aberrant glycosylation and cancer biomarker discovery: a promising and thorny journey. Clin Chem Lab Med.

[21] Ferreira JA, Magalhães A, Gomes J (2017). Protein glycosylation in gastric and colorectal cancers: Toward cancer detection and targeted therapeutics. Cancer Lett.

[22] Kildusiene I, Dulskas A, Smailyte G (2024). Value of combined serum CEA, CA72-4, and CA19-9 marker detection in diagnosis of colorectal cancer. Tech Coloproctol.

[23] Zhan Y, Wang S, Yuan Z (2023). The stool syndecan2 methylation test is more robust than blood tests for methylated septin9, CEA, CA19-9 and CA724: a diagnostic test for the early detection of colorectal neoplasms. Transl Cancer Res.

[24] Chen Y, Ye X, Escames G (2023). The NLRP3 inflammasome: contributions to inflammation-related diseases. Cell Mol Biol Lett.

[25] Wang L, Hauenstein AV (2020). The NLRP3 inflammasome: Mechanism of action, role in disease and therapies. Mol Aspects Med.

[26] Xu X, Wu X, Yue G (2023). The role of Nod-like receptor protein 3 inflammasome activated by ion channels in multiple diseases. Mol Cell Biochem.

[27] Li DL, Ma LL, Guan ZA (2025). Establishment and validation of a clinical prediction model for colorectal adenoma risk factors. Oncol. Lett.

[28] Feng Z, Lin H, Yang X (2023). Diagnostic Value of Inflammation-Related Indicators in Distinguishing Early Colon Cancer and Adenomatous Polyps. Cancer Control.

[29] Qian X, Wang H, Ren Z, Jin F, Pan SY (2021). The value of NLR, FIB, CEA and CA19-9 in colorectal cancer. Zhonghua Yu Fang Yi Xue Za Zhi.

[30] Avuthu N, Guda C (2022). Meta-Analysis of Altered Gut Microbiota Reveals Microbial and Metabolic Biomarkers for Colorectal Cancer. Microbiol Spectr.

[31] Gonzalez RS, Raza A, Propst R (2021). Recent Advances in Digestive Tract Tumors: Updates From the 5th Edition of the World Health Organization "Blue Book". Arch Pathol Lab Med.

[32] Yeung CA (2014). Oral health surveys: Basic methods, 5th edition. British Dental Joural.

[33] Sullivan BA, Noujaim M, Roper J (2022). Cause, Epidemiology, and Histology of Polyps and Pathways to Colorectal Cancer. Gastrointest Endosc Clin N Am.

[34] Mezzapesa M, Losurdo G, Celiberto F (2022). Serrated Colorectal Lesions: An Up-to-Date Review from Histological Pattern to Molecular Pathogenesis. Int J Mol Sci.

[35] Rebecca LS, Kimberly DM, Ann GS (2020). Colorectal cancer statistics, 2020. CA Cancer J Clin.

[36] Ahmad R, Singh JK, Wunnava A, Al-Obeed O, Abdulla M, Srivastava SK (2021). Emerging trends in colorectal cancer: Dysregulated signaling pathways (Review). Int J Mol Med.

[37] Feng CH, Zhang Q, Chen J (2023). Factors influencing age at onset of colorectal polyps and benefit-finding after polypectomy. Medicine (Baltimore).

[38] Walter E, Scott M (2017). The life and work of Rudolf Virchow 1821-1902: "Cell theory, thrombosis and the sausage duel". J Intensive Care Soc.

[39] Hedrick CC, Malanchi I (2022). Neutrophils in cancer: heterogeneous and multifaceted. Nat Rev Immunol.

[40] Zhou WW, Chu YP, An GY (2017). Significant difference of neutrophil-lymphocyte ratio between colorectal cancer, adenomatous polyp and healthy people. Eur Rev Med Pharmacol Sci.

[41] Kim JH, Cho KI, Kim YA, Park SJ (2017). Elevated Neutrophil-to-Lymphocyte Ratio in Metabolic Syndrome Is Associated with Increased Risk of Colorectal Adenoma. Metab Syndr Relat Disord.

[42] Chen W, Xin S, Xu B (2022). Value Research of NLR, PLR, and RDW in Prognostic Assessment of Patients with Colorectal Cancer. J Healthc Eng.

[43] Uçmak F, Tuncel ET (2016). Relationship Between Lesions in Adenomatous Polyp-Dysplasia-Colorectal Cancer Sequence and Neutrophil-to-Lymphocyte Ratio. Med Sci Monit.

[44] Emir S, Aydin M, Can G (2015). Comparison of colorectal neoplastic polyps and adenocarcinoma with regard to NLR and PLR. Eur Rev Med Pharmacol Sci.

[45] Huang C, Liang W, Sun Y (2024). The role of BMI, serum lipid profile molecules and their derivative indexes in colorectal polyps. Adv Lab Med.

[46] Tian Y, Wang K, Li J (2015). The association between serum lipids and colorectal neoplasm: a systemic review and meta-analysis. Public Health Nutr.

[47] Liu B, Wen P, Gu X, Weng R, Liu S (2020). Elevated serum triglyceride predicts recurrence of colorectal polyps in patients with advanced adenomas. Lipids Health Dis.

[48] Zhang R, Yin J, Huo C (2022). The Relationship Between Colorectal Polyps and Serum Lipid Levels: A Systematic Review and Meta-analysis. J Clin Gastroenterol.

[49] Chung YW, Han DS, Park YK (2006). Association of obesity, serum glucose and lipids with the risk of advanced colorectal adenoma and cancer: a case-control study in Korea. Dig Liver Dis.

[50] Tabuchi M, Kitayama J, Nagawa H (2006). Hypertriglyceridemia is positively correlated with the development of colorectal tubular adenoma in Japanese men. World J Gastroenterol.

[51] Wang X, Zou Y, Zhang R (2022). The relationship between serum lipid levels and colorectal serrated lesions: A systematic review and meta-analysis. Front. Physiol.

[52] Kim NH, Lee MY, Park JH (2017). Serum CEA and CA 19-9 Levels are Associated with the Presence and Severity of Colorectal Neoplasia. Yonsei Med J.

[53] Senthakumaran T, Moen A, Tannæs TM (2023). Microbial dynamics with CRC progression: a study of the mucosal microbiota at multiple sites in cancers, adenomatous polyps, and healthy controls. Eur J Clin Microbiol Infect Dis.

[54] Conde-Pérez K, Aja-Macaya P, Buetas E (2024). The multispecies microbial cluster of Fusobacterium, Parvimonas, Bacteroides and Faecalibacterium as a precision biomarker for colorectal cancer diagnosis. Mol Oncol.

[55] Datorre JG, Dos RM, de Carvalho AC (2024). Enhancing Colorectal Cancer Screening with Droplet Digital PCR Analysis of Fusobacterium nucleatum in Fecal Immunochemical Test Samples. Cancer Prev Res (Phila).

[56] Tito RY, Verbandt S, Aguirre VM (2024). Microbiome confounders and quantitative profiling challenge predicted microbial targets in colorectal cancer development. Nat Med.

[57] Crost EH, Coletto E, Bell A, Juge N (2023). Ruminococcus gnavus: friend or foe for human health. FEMS Microbiol Rev.

[58] Hayase E, Hayase T, Mukherjee A (2023). Bacteroides ovatus alleviates dysbiotic microbiota-induced intestinal graft-versus-host disease. Res Sq.

[59] Mandal RK, Mandal A, Denny JE, Namazii R, John CC, Schmidt NW (2023). Gut Bacteroides act in a microbial consortium to cause susceptibility to severe malaria. Nat Commun.

[60] Fuse Y, Ohdaira H, Kamada T (2022). Acute respiratory distress syndrome due to sepsis caused by Bacteroides ovatus after acute appendicectomy. Surg Case Rep.

[61] Ezeji JC, Sarikonda DK, Hopperton A (2021). Parabacteroides distasonis: intriguing aerotolerant gut anaerobe with emerging antimicrobial resistance and pathogenic and probiotic roles in human health. Gut Microbes.

[62] Cui Y, Zhang L, Wang X (2022). Roles of intestinal Parabacteroides in human health and diseases. FEMS Microbiol Lett.

[63] Gunalan A, Biswas R, Sridharan B, Elamurugan TP (2020). Pathogenic potential of Parabacteroides distasonis revealed in a splenic abscess case: a truth unfolded. BMJ Case Rep.

[64] Yawen Z, Xiangyun C, Binyou L (2022). The dynamic landscape of parasitemia dependent intestinal microbiota shifting and the correlated gut transcriptome during Plasmodium yoelii infection. Microbiol Res.

[65] Son HJ, Sohn SH, Kim N (2019). Effect of Estradiol in an Azoxymethane/Dextran Sulfate Sodium-Treated Mouse Model of Colorectal Cancer: Implication for Sex Difference in Colorectal Cancer Development. Cancer Res Treat.

[66] Zaki MH, Lamkanfi M, Kanneganti TD (2011). The Nlrp3 inflammasome: contributions to intestinal homeostasis. Trends Immunol.

[67] Ahechu P, Zozaya G, Martí P (2018). NLRP3 Inflammasome: A Possible Link Between Obesity-Associated Low-Grade Chronic Inflammation and Colorectal Cancer Development. Front Immunol.

[68] Wang X, Jia Y, Wen L (2021). Porphyromonas gingivalis Promotes Colorectal Carcinoma by Activating the Hematopoietic NLRP3 Inflammasome. Cancer Res.

